# Strategies for Highly Efficient Rabbit Sperm Cryopreservation

**DOI:** 10.3390/ani11051220

**Published:** 2021-04-23

**Authors:** Kazutoshi Nishijima, Shuji Kitajima, Fumikazu Matsuhisa, Manabu Niimi, Chen-chi Wang, Jianglin Fan

**Affiliations:** 1 Center for Animal Resources and Collaborative Study, National Institutes of Natural Sciences, 38 Nishigonaka, Myodaiji, Okazaki 444-8585, Japan; 2 National Institute for Physiological Sciences, National Institutes of Natural Sciences, 38 Nishigonaka, Myodaiji, Okazaki 444-8585, Japan; 3 Department of Physiological Sciences, SOKENDAI (The Graduate University for Advanced Studies), Aichi, Okazaki 444-8585, Japan; 4 Analytical Research Center for Experimental Sciences, Division of Biological Resources and Development, Saga University, 5-1-1 Nabeshima, Saga 849-8501, Japan; kitajims@cc.saga-u.ac.jp (S.K.); matsuf@cc.saga-u.ac.jp (F.M.); 5 Department of Molecular Pathology, Faculty of Medicine, Interdisciplinary Graduate School of Medical Sciences, University of Yamanashi, 1110 Shimokato, Chuo 409-3898, Japan; manabun@yamanashi.ac.jp; 6 Animal Resources Section, Okinawa Institute of Science and Technology Graduate University, 1919-1 Tancha, Onna-son, Kunigami-gun, Okinawa 904-0495, Japan; ChenChi.Wang@oist.jp; 7 School of Biotechnology and Health Sciences, Wuyi University, Jiangmen 529020, China

**Keywords:** rabbit, sperm quality, cryopreservation, animal model, assisted reproductive technology

## Abstract

**Simple Summary:**

The rabbit is a valuable animal for both the economy and biomedical sciences. Therefore, the preservation of many rabbit strains is vitally important. So far, sperm cryopreservation is one of the most efficient ways to preserve rabbit strains because it is easy to collect ejaculate repeatedly from a single male and perform artificial insemination to multiple females. Although this method is widely used, there are still some concerns regarding the cooling, freezing and thawing process of sperms, which markedly affects the quality of preserved sperms. In this article, we will review the progress made during the past years in terms of cryopreservation of rabbit sperms and discuss those factors that would possibly influence sperm damage including freezing extender, cryoprotectant, supplements, and procedures.

**Abstract:**

The rabbit is a valuable animal for both the economy and biomedical sciences. Sperm cryopreservation is one of the most efficient ways to preserve rabbit strains because it is easy to collect ejaculate repeatedly from a single male and inseminate artificially into multiple females. During the cooling, freezing and thawing process of sperms, the plasma membrane, cytoplasm and genome structures could be damaged by osmotic stress, cold shock, intracellular ice crystal formation, and excessive production of reactive oxygen species. In this review, we will discuss the progress made during the past years regarding efforts to minimize the cell damage in rabbit sperms, including freezing extender, cryoprotectants, supplements, and procedures.

## 1. Introduction

Rabbits have been indispensable for human life because they are not only valuable for agriculture but also for biomedical research. Rabbits are widely used as a source of meat, hair and fur, and it is estimated that each year, around 300 million rabbits (and hares) are used in the world [1]. Because of their tame characters, rabbits are also raised as a pet. In addition, rabbits are the most-used animals for antibody production for biomedical research. Furthermore, rabbits are similar to humans in terms of cardiovascular physiology and lipid metabolism, and they play an important role in studying human diseases such as atherosclerosis and hypercholesterolemia [2,3]. Along with the development of genetic engineering, a number of gene-modified rabbits have been established as experimental models of human diseases. In addition to transgenic rabbits produced with the conventional pronuclear microinjection technique, knockout rabbits have been established using CRISPR/Cas9 genome editing technology [4]. These established genetically modified rabbits are rare and valuable and thus it is vitally important to breed and maintain rabbit strains for different purposes and preserve them as bio-resources [5].

There are two major ways to preserve rabbit strains. The common way to maintain a rabbit colony is carried out simply by repeat breeding. However, several difficulties with this method exist including space and cost. In particular, rabbit shows severe inbreeding depression [6,7,8,9], thus a considerable number of rabbits are required to keep a colony. For laboratory rabbits, they are usually housed in strictly controlled conditions in terms of temperature, humidity, illumination and microbiological examinations. Furthermore, living animals have a risk of annihilation or escape in the case of a disaster or accident.

**Figure 1:**
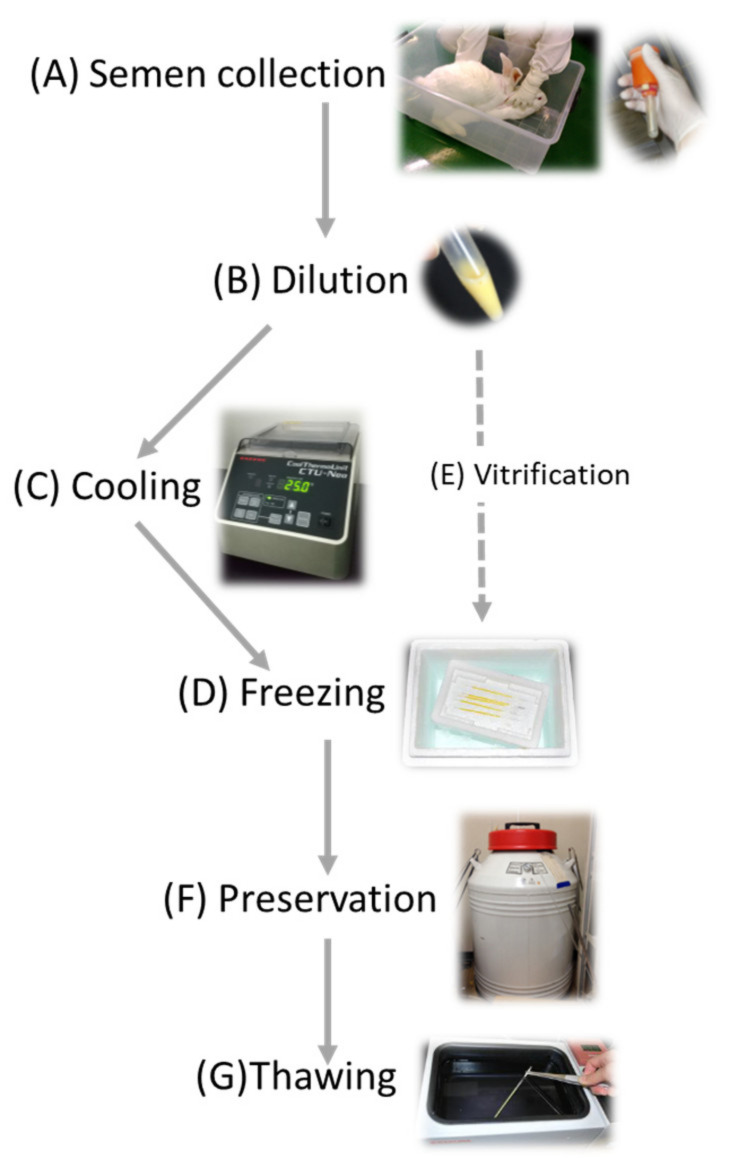
Schematic illustration of the essential process of sperm cryopreservation. (**A**) Semen is collected with an artificial vagina; (**B**) semen is diluted with a freezing extender; (**C**) sperm solution is cooled at slow rate (with a programming incubator); (**D**) sperm solution packed in freezing straws are frozen in vapor of liquid nitrogen; (**E**) vitrification is another option for sperm freezing; (**F**) sperms are cryopreserved in liquid nitrogen, and (**G**) sperms are thawed by immersion in a warm bath.

The second method of maintaining the rabbit colony is the cryopreservation of gametes. Cryopreservation of gametes requires less space and cost than animal breeding. It is generally believed that properly cryopreserved zygotes and gametes can be preserved semi-eternally in a liquid nitrogen tank to keep their fertile and developmental ability. In the case of employing ovum or embryo preservation, ova or embryos are generally obtained with oviduct–uterus dissection from sacrificed females, and a skillful surgical operation is required for the embryo transfer. In contrast, ejected semen can be collected repeatedly without sacrificing males (Figure 1) and artificially inseminated into females can be conducted without specific skills, and thus, sperm cryopreservation would be the first choice for preservation of rabbit strains even though sperms preserved can bring paternal hereditary information only, and immediate offspring is always heterozygosity. However, when concerned with one specific transgene, homozygotes can be obtained in the second generation. Even in the case of livestock animals concerning pedigree related with polygenetic factors, the inbreeding can be avoided by mating live females and cryopreserved sperm with a generation gap.

**Figure 2:**
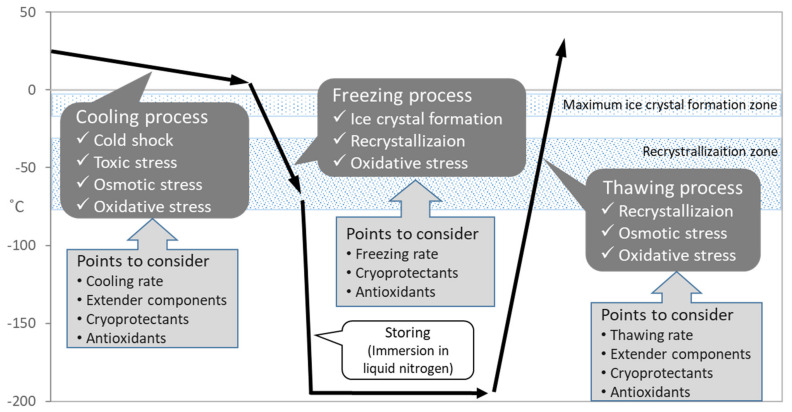
Schematic diagram of temperature changes during sperm cryopreservation process and associated problems.

As mentioned above, the successful preservation of rabbits depends on the efficiency and reliability of procedures in sperm cryopreservation. It is known that the process of sperm cryopreservation, including cooling, freezing and thawing, leads to cellular damage on membrane, cytoplasm and genome structures [10,11] caused by osmotic stress, cold shock, intracellular ice crystal formation, and excessive production of reactive oxygen species (ROS) [12] (Figure 2). During the cooling process, the sperm membrane is injured by cold shock which can be diminished by cooling rate [13] or materials stabilizing the membrane including egg yolk or skim milk [14]. The addition of cryoprotectants causes osmotic and toxic stress, which increases due to prolonged exposure during slow cooling [15]. In following freezing process, major problem is ice crystal formation which grow larger by recrystallization and injures cell [16]. The freezing rate and cryoprotectants application should be considered to diminish the problem. Recrystallization occurs during the thawing process because of entry through the recrystallization temperature zone. Since sperms suffer oxidative stress throughout the cryopreservation process [12], supplementation of antioxidants is considerable for the improvement of sperm quality.

Enormous efforts have been exerted to minimize these detrimental effects, and increase the efficiency and reliability of sperm cryopreservation in the rabbit. In this review, we will discuss recent findings and perspectives including extenders, cryoprotectants, supplements and procedures.

## 2. Effects of Extender and Cryoprotectants

The sperm freezing extenders are commonly composed of a buffer (commonly Tris buffer for rabbit sperms), salt(s) and cryoprotectant(s) to avoid cell damage caused by inadequate pH, osmolality and cryogenic injury [13]. As a component in the freezing extender, egg yolk provides optimal results in rabbit sperm cryopreservation [17,18]. Nevertheless, the egg yolk contains both beneficial and detrimental components for sperms [19,20]. Additionally, there is a sanitary concern in using fresh biotic materials, like the egg yolk, which is subjected to quarantine inspection in the case of import/export. To avoid these risks, lecithin, also known as phosphatidylcholine, a component of egg yolk, is often used to prevent cold shock in sperm cryopreservation [21,22]. It is reported that non-animal originated soybean lecithin with minimal sanitary risks can be used as a substitute for egg yolk based on the motility and fertility of the frozen–thawed rabbit sperm [23]. Skim milk, another substitute, contains advantageous components in the freezing extender in various animal species [24,25,26,27,28,29,30] including rabbits [31]. However, using skim milk in the sperm freezing extender is less common than egg yolk in the rabbit.

The major problem in sperm cryopreservation is the mechanical invasion of sperm cells by ice crystals generated during the freezing process, which decreases the viability of sperm after thawing [32]. Dehydration of cellular water and percolation and immersion of cryoprotectants into the cell are usually performed to avoid the generation of the ice crystals. Both permeable (such as glycerol, dimethyl sulfoxide (DMSO), ethylene glycol, and amides) and non-permeable (saccharides, lipoprotein and Ficoll) are used as cryoprotectants. The permeant agent binds to intracellular free water leading to suppression of the generation of ice crystals. On the other hand, the non-permeant agents surrounding the cell increase extracellular osmotic pressure and enhance dehydration of the cell [33,34,35].

Lots of studies have been conducted regarding concentrations and combination effects of the cryoprotectants on rabbit sperm cryopreservation as encyclopedically reviewed by Mocé and Vicente [14]. Though glycerol and DMSO are the most common permeable cryoprotectant, it was suggested that glycerol is not suitable for rabbit sperm cryopreservation [14] possibly because of its low water permeability and high activation energy [36]. Additionally, it is known that high concentrations of DMSO show adverse effects on sperm quality in terms of motility and acrosome integrity [37,38]. Amides, namely lactamide and acetamide, are another candidate as a permeable cryoprotectant for rabbit sperm cryopreservation [39,40] and provide better results than glycerol or DMSO [41,42].

Lactose, sucrose, maltose, raffinose, treharose, and dextrans are used as non-permeable cryoprotectants in sperm cryopreservation, and have been shown to have an effect of stabilizing the plasma membrane during freeze–thaw process by interacting with membrane phospholipids [33,34,35] in addition to increasing the osmotic pressure.

## 3. Effects of Freezing and Thawing Procedure

Two principal techniques, slow freezing and vitrification, were employed for sperm freezing (Figure 1). During the freezing process of cells, extracellular water is frozen more quickly than intracellular water [42]. Generation of the ice corresponds to the decrement of water, which produces an imbalance of cellular osmotic pressure and intracellular water moving out of the cell. In slow freezing, generation of the intracellular ice crystal is suppressed since the cellular dehydration progresses slowly during a longer time of freezing. Concomitantly, concentrations of solute are deleterious to the cells and exposure time will be increased within a slow freezing procedure [42]. On the other hand, in vitrification, the cells are immediately cooled to −196 °C in liquid nitrogen and exposure time to high concentrated cryoprotectant is minimized. A countermeasure, including the application of high concentrated cryoprotectans, to suppress the ice crystal generation is required since dehydration is not always sufficient in vitrification.

Vitrification procedure has been explored in various mammalian sperm including humans [10,43]. It is known that sperms with larger heads are more susceptible to cold shock [44], which reduces sperm survivability [45]. The head of rabbit sperms is relatively large [32] and no efficient procedure in vitrification has been established. Rosato and Iaffaldano [46] compared the frozen–thawed sperm survival and fertility with/without various cryoprotectants between slow freezing and vitrification, and showed that the outcome from vitrification was far inferior to slow freezing. It is known that cells with a high water permeability show better tolerance for rapid freezing than those with a low water permeability [47], and rabbit sperm shows a low water permeability [36]. Therefore, conventional slow freezing is the prime choice for sperm cryopreservation at the current moment.

In the slow freezing procedure, sperms are cooled down to 5 °C before freezing to avoid the cold shock [45]. In the case of rabbits, Mocé et al. [48] reported that slow cooling to 5 °C improved neither fertility rate nor prolificacy in relation to cryopreservation. On the other hand, Maeda et al. [49] revealed that the viability of frozen–thawed rabbit sperm cooled at −0.1 or −0.2 °C/min (slower) were higher than at −0.8 °C/min. These data indicate that a faster cooling rate negatively affects sperm viability and does not improve reproductive performance after freezing and thawing. Obtaining more viable sperm results in efficiency improvement of cryopreservation, since the possibility of successful fertilization is increased. Additionally, in rabbits, time held at 5 °C affects the quality and reproductive performance of sperm. It was reported that longer holding time (90 min) at 5 °C increases the quality of frozen–thawed rabbit sperm and their fertilizing ability [50], and conversely, and shortened holding time (10 min) decreases it [51].

The ice crystal formation during the freezing process damage cells as described above and freezing protocol is also one of the concerns. In rabbit sperms, it was revealed that sperms frozen at slow (−15 °C/min) and fast (−60 °C/min) rates were lower in the quality and fertilizing ability than those frozen at medium (−40 °C/min) rate and in static liquid nitrogen vapor [52].

The thawing rate of frozen sperms is also known to affect the quality of sperms. Though frozen sperms are usually thawed at temperatures close to body temperature, it is generally recognized that high thawing rates provide better results [32,53]. It is possible that low thawing rates enhance recrystallization, a phenomenon that relatively many small ice crystals aggregate and form fewer larger ice crystals, which causes more severe damage to the cells [16]. Though thawing temperatures over 60 °C were adopted in some cases [54,55,56,57], exposing duration to a high temperature must be strictly controlled. Mocé et al. [58] compared the fertility rate and prolificacy of frozen rabbit sperm between thawed at 50 °C and 70 °C for 10–12 s, and concluded that thawing at 50 °C provided better results. In contrast, Chen and Foote [59] reported that the mortality of sperm thawed at 25 °C for 1 min was superior to those thawed at 45 °C for 30 s or 65 °C for 7 s following mechanical seeding at −6 °C. Therefore, appropriate thawing temperature can be affected by other conditions including cooling or freezing procedures, and further studies are demanded.

## 4. Cryopreservation Device

The choice of the freezing devices affects the quality of frozen–thawed sperm. Glass vials [31], plastic ampoules, polyvinylchloride tubing, [60] and pellet [61] addition to straws, which are widely employed in recent years, have been utilized for rabbit sperm cryopreservation. Thermal conductance to sperm depends on the shape, size and material of the device, which influences cooling, freezing and thawing rates. It was shown that rapid warming had a more dominant effect on survival than rapid cooling in mouse oocyte [62], and rapidly warming to the critical temperature range (−70 °C to −35 °C) at which intracellular ice is likely to form by recrystallization would improve cryopreservation efficiency of cell [63]. In the case of rabbit sperms, though the rapid freezing method (vitrification) has not been commonly utilized, the development of a new method and device for rapid thawing could lead to a great improvement of sperm cryopreservation efficiency.

## 5. Effects of Supplements

As mentioned above, lots of studies have been performed to address components of the sperm freezing extenders; however, results were not always satisfying. Some additional supplements can improve the efficiency in sperm cryopreservation (Table 1).

The antioxidant is one of the most expected supplements for sperm cryopreservation by eliminating the excessive production of ROS [12]. Zhu and colleague investigated the effects of supplementation of amin E analogue [64], cysteine [65], glutamine [66], trehalose [67] and melatonin [68], and revealed that supplementation of these antioxidative agents in Tris-citrate-glucose extender decreased ROS levels and improved the quality of frozen–thawed rabbit sperms. Fadl et al. confirmed 1.0 mM melatonin improved the motility, viability, membrane and acrosome integrities, and DNA integrity of frozen–thawed rabbit sperms in different extender (INRA-82) [69] Additionally, curcumin and curcumin nanoparticles were confirmed to improve the post-thawed quality of rabbit sperms via redox signaling and reduce the apoptosis process [70]. On the other hand, Maya-Soriano et al. reported that supplementation of bovine serum albumin, retinol and retinyl in the sperm freezing extender has no beneficial effect on the viability, mortality, progressivity, and acrosome and morphological integrity of frozen–thawed rabbit sperms at the concentrations they tested [71]. In this sense, other researchers have revealed the importance of the antioxidant concentration to achieve beneficial effects on rabbit sperm quality after thawing. Thus, while 4 mM of glutathione (GSH) improved [72] the viability, mortality, progressivity and acrosome integrity of frozen–thawed rabbit sperms, 0.5 mM of GSH did not provide similar results [73]. More studies are necessary for the adjustment of the freezing media in this species.

There are also some supplements with controversy surrounding their effects on rabbit sperm cryopreservation. Bovine serum albumin (BSA) is known to have a dual effect to protect sperm from osmotic stress by increasing membrane resistance [12] and oxidative stress by trapping free radicals [74]. Therefore, supplementation of BSA has been shown to improve the quality of frozen–thawed sperms in some species [75,76,77,78]. However, in rabbits, controversial results regarding the usage of BSA have been reported. Thus, while Maya-Soriano et al. [71] did not find any beneficial effect on sperm viability, mortality, progressivity, and acrosome and morphological integrity, Rosato and Iaffaldano [46] showed that frozen–thawed rabbit sperm display better mortality and DNA integrity when BSA is combined with sucrose or trehalose. These facts suggest that concentration and/or combination with other contents of the extender are important for exerting the beneficial effects of BSA. Moreover, gelatin is known to have protective effects including reduction of the sperm sedimentation and maintenance of the pH homogeneity in cooled semen [79] which has been confirmed in rabbits as well [80,81]. However, Cortell et al. [82] reported that gelatin addition did not improve the motility and viability nor fertility and prolificacy of frozen–thawed sperms in the rabbit. In later years, it was reported adding 2% gelatin enhanced the freezability and fertility of frozen–thawed rabbit sperms [83].

Antifreeze proteins (AFPs) are known to stabilize cell membranes and inhibit ice crystal growth and ice recrystallization [84,85]. With such functions, supplementation of AFP type III in the sperm freezing extender can improve the quality of frozen–thawed rabbit sperms [86]. However, AFPs are not easy to extract from natural resources such as fungi, bacteria, plants, insects and fish that are adapted to cold environments [87] and AFPs derived from other organisms can be detrimental to sperms or inseminated female animals. Tekin and Daşkın [88] utilized polyvinyl alcohol (PVA) instead of AFPs for ice recrystallization inhibition and showed that supplementation enhances motility, viability, acrosome integrity and mitochondrial activity in frozen–thawed rabbit sperms.

By stabilization of cell membrane, supplementation of a silk protein, sericin [89] and cholesterol-loaded cyclodextrin [90] improved motility and quality of frozen–thawed rabbit sperm. However, since these supplements are known to inhibit acrosome reaction [89,91], further studies on enhancing its fertility are required for practical use.

## 6. Another Preservation Strategy

The freeze-drying technique is an alternative technology for the long-term preservation of sperms [92]. Liu et al. [93] showed that freeze-dried rabbit sperms maintain the ability for full-term development in spite of immobilization, membrane breaking, and tail fragmentation. It is important to store freeze-dried sperms at low temperatures for stable, long-term preservation, and properly stored freeze-dried sperms maintain their fertility for years [92]. It is necessary to operate intracytoplasmic sperm injection (ICSI) [94] and embryo transfer, which require a skillful technique and particular device for using the freeze-dried sperms. One of the advantages of sperm preservation in rabbits is the applicability of artificial insemination as mentioned above, and preservation of freeze-dried sperms would be just a spare option in rabbit sperm preservation.

## 7. Conclusions and Perspectives

As stated above, numerous studies on improving rabbit sperm cryopreservation have been conducted from various aspects including freezing procedure, type, concentration and combination of cryoprotectants. In spite of this, a standard procedure for rabbit sperm cryopreservation has not been well established due to various and irreproducible results from each study.

One of the reasons for the irreproducibility in rabbit sperm cryopreservation may be derived from the differences in sperm conditions. It is known that freezabilities of rabbit sperms differ among individuals [95] or breeds [96]. However, rabbit breeds used in some reproductive studies have not strictly and well defined rather than other livestock animals such as cattle, horses or pigs, and some authors even did not provide enough information about the rabbit breed examined. There are several reports which indicate associations between the sperm freezability and abundance of particular components in seminal plasma including proteins and fatty acids in some species [97,98,99,100,101,102]. The individual and breed difference in sperm freezability can be explained by such seminal plasma traits. It would be possible to improve the sperm freezability and resolve the individual or breed differences by complemental supplementations according to the seminal plasma trait. In the rabbit, it was revealed that genotype, i.e., breed, affects the abundance of some seminal plasma proteins [103], which are associated with sperm quality [104]. Furthermore, there is no information about the association between sperm freezability and seminal plasma traits in rabbits, and further studies are needed. Again, lack of information about the rabbit breed can disturb the improvement of rabbit sperm cryopreservation efficiency.

Another possible reason for the irreproducibility is disunified evaluation criteria of the quality of the sperms among the reports. Some studies report both the quality of the frozen–thawed sperms and their fertility and prolificacy, and others do only one of them. Examination of fertility and prolificacy involves artificial insemination procedures which affect the results of the study. On the other hand, fertility and prolificacy cannot be estimated by the sperm quality alone, even though obtaining more motile sperms is generally advantageous for efficient reproduction [105,106,107]. Additionally, the rate of rapid and progressive motile sperm would be important for successful artificial insemination, since the inseminated sperm need to reach the ova via a long reproductive tract [108,109,110].

In any case, to achieve a consensus on the efficient method for rabbit sperm cryopreservation, extensive investigations are required under unified evaluation criteria and conditions except for factors like extenders, cryoprotectants and procedures to be examined. On the other hand, it seemed that the supplements like antioxidants generally just add their effects without interfering with other components in the extender. Therefore, most of the supplements exert expected effects on the quality of frozen–thawed rabbit sperm even in various conditions including the extender (Table 1). The dose and combination of the supplements can be a key subject for highly efficient rabbit sperm cryopreservation in future studies.

As Dr. Robert G. Edwards was awarded The Nobel Prize in Physiology or Medicine for the development of in vitro fertilization (IVF) in 2010, assisted reproductive technology (ART) including IVF is an indispensable medical procedure in this modern age. The rabbit is also known as a prime reproductive model for human health, because of (1) exact staging of early embryonic developmental and maternal pregnancy stages, (2) large-sized blastocysts amenable to micromanipulation, (3) cell-lineage-specific analyses, (4) gastrulation stages representative of mammalian development, and (5) placental morphology and function similar to the human [111]. Therefore, the development of reproductive technology in rabbits, which leads to the improvement of medical procedures in humans, is very important and desirable.

## Figures and Tables

**Table 1:** Effects of supplements on quality of thawed rabbit sperm frozen by slow freezing method.

		Effective Concetration	Sperm Quality	EXtender Additives	Dilution Rate (Sperm:Extender)	Referrence
Antioxdants						
	Trolox (vitamine E analogue)	200 μM	Improved	4% DMSO 20% egg yolk	1:1	[64]
	Cysteine	5, 7.5 mM	Improved			[65]
	Glutamine	20 mM	Improved			[66]
	Trehalone	100 mM	Improved			[67]
	Melatonin	0.1 mM	Improved			[68]
		1.0 mM	Improved	INRA−82 (0.15% skim milk)	1:1	[69]
	Oxidised glutathione	0.5 mM	No effect	3.5 M DMSO 0.1 M sucrose	1:1	[73]
	Reduceed glutathione	0.5 mM	No effect			
		4 mM	Improved			[72]
	Curcumin nanoparticles	1.5 μg/mL/0.3	Improved	7% glycerol 20% egg yolk	1:4	[70]
	Retinol	50, 100, 200 μM	No effect	Gent B^®^ * (egg yolk, glycerol)	1:2	[71]
	Retinyl	0.282, 2.82 μg/mL	Deteriorated			
Bovine serum albumin		5, 30, 60 mg/mL	No effect			
		0.5%	Improved	10% DMSO 0.1 M sucrose/0.1 M treharose	1:1	[46]
Gelatin		2%	No effect	3.5 M DMSO 0.1 M sucrose.	1:1	[82]
		2%	Improved	2% glycerol 10% egg yolk	1:5	[83]
Antifreeze protein III		0.1, 1 mg/mL	Improved	20% egg yolk	1:5	[86]
Polyvinyl alcohol		0.001, 0.01, 0.1, 1, 2 %	Improved	5 ^a^, 4 ^b^, 3 ^c^, 3 ^d^, 2 ^e^% glycerol 5 % egg yolk	N/A	[88]
Sericin		0.1%	Improved	16% Me_2_SO 2% sucrose	1:2	[89]
Cholesterol-loaded cyclodextrin		1 mg/40 × 10^6^ sperm	Improved	6% acetamide 20% egg yolk	1:5	[90]

*: Minitüb, Tiefenbach, Germany. Concetrations of glycerol when supplemented with a: 0.001, b: 0.01, c: 0.1, d: 1, e: 2% of polyvinl alchool.
